# CCSchool: a multicentre, prospective study on improving continuum of care in children and adolescents with mental health problems associated with school problems in Germany

**DOI:** 10.1186/s12913-018-3713-6

**Published:** 2018-12-06

**Authors:** Isabel Boege, Julia Herrmann, Julia Katharina Wolff, Ulrike Hoffmann, Michael Koelch, Marc Kurepkat, Steffen Lütte, Alexander Naumann, Hans Dieter Nolting, Jörg Michael Fegert

**Affiliations:** 1 CAP, ZfP Suedwuerttemberg, Weingartshoferstrasse 2, 88214 Ravensburg, Germany; 2 CAP, Universitaet Ulm, Ulm, Germany; 3 CAP, Psychiatrische Klinik Lueneburg, Lueneburg, Germany; 4 CAP, Ruppiner Kliniken, Brandenburg Medical School, Neuruppin, Germany; 5 Clinische Studien Gesellschaft (CSG), Berlin, Germany; 6 IGES Institute, Berlin, Germany

**Keywords:** Mental health problem, Adolescent, Child, Medical health services, School based intervention, Access to psychiatric care, School problem

## Abstract

**Background:**

Most psychiatric disorders in childhood and adolescence cause impairment in academic performance. Early interventions in school are thought to reduce the burden of disorder and prevent chronicity of disorder, while a delay in reachable help may result in more severe symptoms upon first time presentation, often then causing upon first-time presentation immediate need of inpatient care.

**Methods:**

The study aims at reducing hospitalization rates and increasing social participation and quality of life among children and adolescents by establishing collaborations between schools, mental health care services and youth welfare services. CCSchool offers children and adolescents, aged six to 18 years, who present with psychiatric problems associated to school problems, a standardized screening and diagnostic procedure as well as treatment in school if necessary. Students can participate in CCSchool in three federal states of Germany if they a) show symptoms vindicating a mental health diagnosis, b) present with confirmed school problems and c) have a level of general functioning below 70 on the children global assessment of Functioning (C-GAF). Intervention takes place in three steps: module A (expected *n* = 901, according to power calculation) with standardized diagnostic procedures; module B (expected *n* = 428) implies a school-based assessment followed by a first intervention; module C (expected *n* = 103) offering school-based interventions with either four to six sessions (basic, 80% of patients) or eight to 12 sessions (intensive, 20% of patients).

Primary aim is to evaluate the effectiveness of CCSchool, in reducing the need of hospitalization in children with mental health problems. The analyses will be conducted by an independent institute using mainly data collected from patients and their caregivers during study participation. Additionally, claims data from statutory health insurances will be analysed. Relevant confounders will be controlled in all analyses.

**Discussion:**

Evaluation may show if CCSchool can prevent hospitalizations, enhance social participation and improve quality of life of children and adolescents with mental health problems by providing early accessible interventions in the school setting.

**Trial registration:**

Deutsches Register Klinischer Studien, Trial registration number: DRKS00014838, registered on 6th of June 2018.

## Background

Children and adolescents with any kind of disability, including children and adolescents with mental health disabilities, need good medical care all their life. Prevalence of psychiatric disorders in childhood and adolescence is high, ranging between 10 and 20% [1, 2]. More than 50% of these children and adolescents remain mentally impaired in adulthood [3–5, 6]. Once a severe mental health disease occurs, it tends to become chronic and may need repeated hospitalizations affecting daily life and social integration. It has been shown that mental health problems in children and adolescents subsequently not only contribute to lower achievements in education but bear an increased rate of health risk behaviour such as self-harm or suicidal behaviour [7] – issues that have a huge impact on later quality of life in adulthood. Thus, early diagnosis and adequate treatment in childhood mental health disorders are crucial for prevention of future persistence of disease. However, children and adolescents often experience considerable barriers accessing mental health services (e.g. lack of knowledge of mental health problems among care givers, difficult referral procedures, long distances and lack of transportation, especially in rural areas) [8, 9].

As children usually spend a considerable amount of time in school, schools have been referred to in the past as “the primary health care system for children” [10]. Hence, it is not surprising that in 70% of the cases schools indicate problematic behaviour and become thereby for the children and adolescents the main gate of entry into the mental health care system [11, 12]. Once having been seen by a psychiatrist early interventions in school may reduce the burden of disorder and help prevent chronicity of mental health problems, while on the other hand a delay in help may result in more severe symptoms when the child/adolescent finally presents. It is therefore of high importance to develop, implement and evaluate possible easily accessible pathways for provision of psychiatric care for children and adolescents with mental health problems.

Offering a low threshold school-based intervention in schools may promote a positive attitude towards mental health treatment among children and adolescents [13]. School may therefore not only be the ideal place for early detection of disease but also should be the place for early professional intervention [14]. Evidence from countries where school-based interventions have already been implemented, shows that relevant programs yield significant positive effects on students’ emotional well-being and behavior [7]. Also additional benefits in the treatment of children with mental health problems have been shown for treatments combining parental training with child or adolescent group-cognitive-behavioural-therapy (CBT) in school [6]. Cossu et al. [15] summarized in a review that school-based intervention programs can reduce symptoms of almost all mental disorders. In a recent study by Sanchez et al. [16], school-based services yielded a small-to-medium effect in decreasing mental health problems. The largest effects were found for targeted interventions, followed by selective prevention. Mental health services targeting externalizing problems were shown to be most effective when they were integrated in students’ regular academic schedule [16].

However, in the past decades, continuum of care between the school system, youth welfare system and mental health care services have not been existent in Germany. Reimbursement for psychiatric outreach work was not provided. In the absence of intensive outreach programs, rates of admission to inpatient services among children and adolescents in need of intensive psychiatric treatment were then rather high, while thresholds to use inpatient programs were inadequately lowered, alternative treatment options are lacking [17, 18]. Between 2007 and 2014 inpatient admissions in child and adolescent psychiatry in Germany increased by 38%, while the average duration of stay reduced only by 14%, resulting in an overall increase of inpatient beds needed by 17%. As a consequence transfer of inpatient-treatment effects to school and family surroundings is difficult, diminishing the chances for long-term effects and leading to increasingly often costly and frustrating “revolving door effects” with patients moving back and forth between in- and outpatient settings. On the other hand mental health treatments in natural surroundings (school, family) have been shown to acchieve more stable treatment effects over time. In those settings, relevant problems can be treated where they arise, involving school and family members alike [19–23].

On the 1st of January 2017 the “Act for further development of healthcare and reimbursement of psychiatric and psychosomatic services” (PsychVVG – “Gesetz zur Weiterentwicklung der Versorgung und der Vergütung für psychiatrische und psychosomatische Leistungen”) came into effect in combination with § 115d [24] in Volume V of the German Social Security Code (SGB V). A milestone in provision of care has been laid and the tide has turned: It is now possible for hospitals in Germany to offer continuum of care in form of outreach treatments to patients with mental health issues [25].

CCSchool aims to implement and evaluate a comprehensive, innovative outreach program, which - if effective - may later find its’ way into routine care. CCSchool focusses on offering early diagnostic and if necessary school-based interventions in a standardized procedure. The evaluation of CCSchool will answer the following research questions by comparing patients, who take part in CCSchool (intervention group), to patients in standard care (control group):

1. Will - once continuum of care has been established in schools - the number and duration of inpatient and day-patient treatments in children and adolescents with mental health problems that manifest in school decrease?
2. Will participation and integration of a child/adolescent with mental health issues and school problems improve when offering an adequate treatment on site (within schools)?
3. Will quality of life for the patient and family be improved significantly in CCSchool intervention groups?

CCSchool is supported by a grant from the Innovation funds of the Federal Joint Committee of the Federal Republic of Germany under the number 01NVF17020. The Study has been registered in the German Clinical Trials Register under DRKS00014838 and was approved by the ethics committee of the University of Ulm on the 4th of August 2017 (covering the study sites: Ravensburg, Ulm, Neuruppin) According to state regulations of Lower Saxonia a separate ethics approval was needed for the study site Lueneburg, which was obtained on the 25th of October 2017 by the ethics committee of Lower Saxonia.

## Methods/design

CCSchool comprises the following three modules as part of a stepped-care model:

- Module A: standardized diagnostic procedure,
- Module B: School-based assessment followed by a first intervention (therapeutic assessment)
- Module C: School-based treatment.

There are two kinds of therapists working on one case: Responsible Therapists (RT) and CCSchool-Therapists (CCST).

The RT manages the case and decides which patients enter module A and may receive the treatment of the modules B and C in addition to module A. The role of the RT can be assumed by physicians, psychologists and other mental health professionals specializing in child and adolescent psychiatry/psychotherapy or paediatrics.

Eligible as CCSchool-Therapist (CCST) are occupational therapists, psychologists in training, social workers, nurses and other health professionals. The CCST will conduct the school-based assessment and the school-based interventions (modules B and C).

All of the participating therapists (RTs and CCSTs) have to complete an e-learning programme before being eligible to recruit or work with patients in CCSchool.

Students suspected of mental health problems associated with school problems, will undergo a screening for CCSchool. Students can be referred to the screening procedure either by (a) parents, (b) a paediatrician/child and adolescent psychiatrist/GP.

Inclusion criteria are

- a psychiatric diagnosis and
- school problems (academic or social) pointed out by the teacher or parents or high scores on the school-items of the Columbia Impairment Scale (CIS) filled-in by parents and/or students and
- a psychosocial functioning level on the Children Global Assessment of Functioning Scale (C-GAF) equal or below 70.

Exclusion criteria are: primary diagnosis of substance abuse or an issue of child protection that has to be addressed first.

Once found eligible for CCSchool, the parents and the child or adolescent will be informed about the study, and, if they are interested in participation, parents and child/adolescent will be asked to sign an informed consent form.

CCSchool will take place in about 20 administrative districts (with an average of 150.000 residents per district), which are evenly randomized into districts for recruitment of the intervention group and districts for recruitment of the control group. Not all health statutory insurances wanted to participate, resulting in about 60% of the children and adolescents in school being eligible for CCSchool. If a child is screened positive and is covered by an health statutory insurance which does not participate in general, the child can enter the contract individually and thereby become eligible for CCSchool.

Assuming hospitalization rates in the eligible population are about five times higher than national average due to the high selectivity (about 5.810 cases per 100.000 children and adolescents), the expected effect of CCSchool treatment will lead to a decline in admissions by 10% (which would then be 5.230 cases per 100.000 children and adolescents). A corresponding power analysis showed that the expected difference can be found when including 20 clusters (districts), with an average cluster-size of 90 patients and a coefficient of variation of k = 2.6 with a power of 90% and an alpha of 5%. Thus, CCSchool plans to recruit about 900 participants in the intervention and control group, respectively, who participate at least in module A. About 50% of subjects are expected to participate in module B and another 30% will be recommended to receive treatment in module C. According to power calculation, a sample of about 900 participants in each group is sufficient to find the expected effects.

### Intervention group

If the patient attends a school that is located in an intervention group district, the following modules will be offered according to a stepped-care concept:

#### Module A

Module A is administered by the RT and comprises (1) a physical examination, (2) a standardised psychological assessment with the following questionnaires:

- Strengths and Difficulties Questionnaire (SDQ),
- Child Behaviour Checklist (CBCL),
- Youth Self Report Form (YSR) for children aged > 11 y

and (3) evaluation of possible participation deficits, systematically assessed by conducting a half-standardized interview with the parents.

The diagnostic findings of module A will be summarized in a detailed clinical report. If the assessment result confirms a mental health diagnosis associated with manifest school problems and participation deficits the child or adolescent enters module B.

#### Module B

Module B includes (a) a school-based assessment as well as (b) a first intervention. The school-based assessment is carried out by the CCST, who observes the student in school assessing his/her social behaviour, ability to stay focussed, his/her learning behaviour in class as well as his/her behaviour during recess. The CCST fills in a standardized school assessment form based on these observations. The class-teacher will be asked to fill in the teacher report form (TRF), and the CCST will conduct a half-standardized interview with the class-teacher.

Once school assessment took place, the RT and CCST will invite the family, patient and possibly teacher to the office to discuss the results and offer therapeutic assessment (TA, a method by Ougrin et al., [26]) as first intervention. TA consists of a joint construction of a diagram (based on a cognitive analytic therapy paradigm) that consists of three elements: reciprocal roles, core pain and maladaptive behavioural patterns, which are each assumed to play a crucial role in setting off and promoting dysfunctional cycles of behaviour. The target problem is identified jointly. Motivation for change is considered and enhanced and then potential “exits” (i.e. ways of braking the vicious cycle of school failure/problems) are installed.

One week after TA, the RT sends an “understanding letter” to the child/adolescent, enhancing the identified alternative “exits”. Four weeks after TA the RT calls the family to ask if change has taken place. If this is not the case the child or adolescent will be offered module C.

#### Module C

Module C is offered in two forms:

- regular CCSchool-treatment (about 80% of subjects in module C should qualify for this setting): with four to six sessions in school within 3 months,
- intensive CCSchool-treatment (about 20% of subjects in module C should qualify for this setting) with 6 to 12 sessions in school within 3 months as well as optional phone calls between the sessions.

Goal attainment scaling will be used to monitor the process [27]. The methods of intervention to be used are not manualized. Instead the RT and CCST choose individually for each student those methods deemed appropriate for the goal to be reached according to goal attainment scaling. Possible interventions are: psychoeducation of teachers and/or parents, social competence training, conveying learning strategies or methods of staying focussed, installing CBT programs etc. The methods themselves and their effectivity are not the focus of this study and will not be evaluated. These are standard interventions. The query is more so, if the fact that they are applied in school will make a difference.

Assessment of quality of life (KIND-L), level of functioning (C-GAF), school absenteeism and level of impairment (CIS) will take place at four time points: At recruitment (T0), 6 months after recruitment or after completion of last module (which can be A, B or C) (T1), 12 months (T2) and 18 months (T3) after recruitment.

**Fig. 1:**
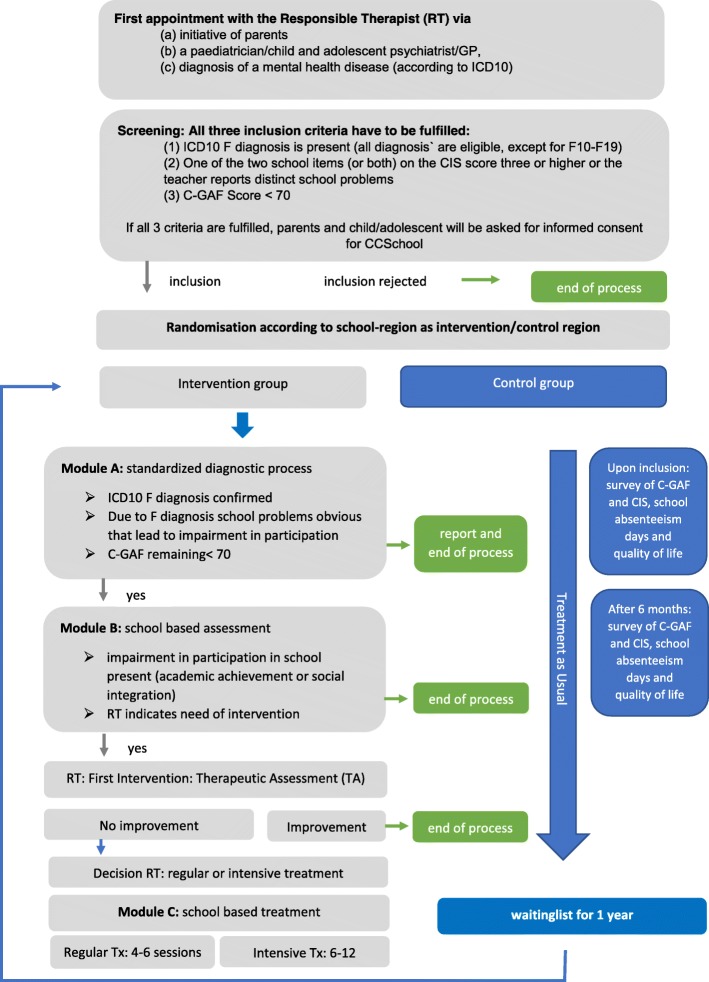
Overview of modules and procedures as part of the CCSchool stepped-care-model

The steps of the study are summarized in Fig. 1.

### Control group

The control group follows a waiting list design. Recruited children and adolescents, who attend school in a control group district, will receive treatment as usual (TAU) for 12 months. Level of functioning (C-GAF), level of impairment (CIS), quality of life (KIND-L) and days of school absenteeism will be recorded at recruitment (T1), as well as after six (T2) and 12 months (T3). After 12 months the parents and child/adolescent will be screened again for eligibility and – if still eligible - offered to enter the intervention part of the study and participate in module A as well as B and C if necessary. After an additional 6 months or after completion of module C the last assessment (T4) will take place.

### Instruments

#### Columbia impairment scale (CIS)

The CIS is a 13-item scale that can be administered by a lay interviewer to either parents or child to provide a global measure of impairment. The 13 items tap four major areas of functioning: interpersonal relations, broad psychopathological domains, functioning in school, and use of leisure time. Items are scored on a spectrum ranging from 0 “no problem” to 4 “a very big problem”. The instrument shows good reliability with Cronbach’s α between 0.70 and 0.89 [28].

#### Children Global Assessment of Functioning (C-GAF)

The Children’s Global Assessment of Functioning (C-GAF) is a practitioner-rated global measure of functioning, which is assessed on a scale from 1 “most impaired” to 100 “superior level of functioning” broken down into 10 equal parts. Shaffer et al. reported an interrater reliability of 0.84 and a test-retest reliability of 0.85 [29].

#### Strengths and Difficulties Questionnaire (SDQ)

The Strengths and Difficulties Questionnaire (SDQ) is a brief behavioural screening questionnaire with 25 items. It exists in a version for youths aged 11–16 years and for parents of 3–16 year olds. The SDQ asks about 25 positive and negative attributes that can be summarized in five subscales: emotional symptoms, conduct problems, hyperactivity/inattention problems, peer relationship problems and prosocial behaviour. Items are scored as “not true”, “somewhat true” and “certainly true” [30].

#### Child behaviour Checklist (CBCL), Teacher Report Form (TRF), and Youth Self-Report Form (YSR)

The CBCL/6–18 consists of 140 items, the TRF of 117 Items and the YSR of 118 Items. Most items are a 3-point likert-scale from 0 (not true (as far as you know)) to 2 (very true or often true). Each form also has different introductory questions that may be rated on a likert scale (with varying numbers of options and anchor points), be open-ended, or multiple-response. The questionnaires can be filled in 10 to 15 min. Subscales that are assessed by summing up items are anxious/depressed, withdrawn/depressed, somatic complaints, social problem, thought problems, attention problems, rule-breaking behaviour, aggressive behaviour. It may also provide information regarding internalizing problems and externalizing problems as well as a total score across all items. The authors of the CBCL report test-retest reliabilities of 0.73–0.94, internal consistency reliabilities (alphas) of 0.63–0.97, and inter-rater reliabilities of 0.57–0.88. For the YSR test-retest reliabilities were 0.67–0.91, and internal consistency reliabilities (alphas) 0.55–0.95. The authors of the TRF report test-retest reliabilities of 0.60–0.96, internal consistency reliabilities (alphas) of 0.72–0.997, and inter-rater reliabilities of 0.20–0.76 [31].

#### KINDL Questionnaire

The KINDL Questionnaire consists of 24 likert-scaled items covering six dimensions: physical well-being, emotional well-being, self-esteem, family, friends and everyday functioning (in school/kindergarten). The six sub-scales can be summed up to a total score. The questionnaire is available as a self report (children/adolescents four to 17 years) and a proxy report version (parents of children between three and 17 years). The scale shows a good reliability (Cronbach’s α > 0.70 for most of the subscales and samples) and a satisfactory validity [32].

### Goals

The main goal of CCSchool, and therefore the primary outcome is to reduce the necessity of inpatient admissions in children and adolescents with mental health and school problems which in consequence may reduce health care costs. In addition, we expect CCSchool to result in increased quality of life as well as social participation in children and adolescents. To achieve these goals early interventions in school will be offered, involving relevant teachers and caregivers into the treatment process. CCSchool comprises the development and evaluation of a model of continuum of care including a collaboration of the mental health system, schools and social services. This model of continuum of care not only results in early treatment by the relevant health professionals of children with mental health problems and impairment in school-participation but also offers intervention in the very place where problems primarily arise: in school.

The implementation of CCSchool requires the establishment of collaboration between the health care and educational systems as well as specific training of therapists via e-learning to use standardized diagnostic procedures and to offer materials for first low-threshold interventions and/or psychoeducation of teachers, parents and children or adolescents.

Secondary outcomes are:

1. Participation in schooling/apprenticeship: Goal: reduction of absenteeism-days from school due; regular school attendance
2. Disease related specific symptoms: Goal: reduction of mental health symptoms with improvement in level of functioning.
3. Quality of life: Goal: improvement of overall quality of life and in particular in the subscale emotional well-being.
4. Feasibility of CCSchool: establishing (a) a standardized diagnostic procedure for children and adolescents with mental health problems associated with school problems and (b) a school-based treatment in the German Health Care System.

### Evaluation and study analyses

An external independent research institute (IGES Institute GmbH) will evaluate the achievement of the endpoints in CCSchool. The evaluation will use primarily data collected and assessed during the study on level of functioning, level of impairment, absenteeism and quality of life as well as data from documentation and process evaluation. In addition, claims data from the statutory health insurances will be analysed.

The study uses a cluster-randomized design with a waiting-list control group. Participating districts will be randomly assigned to an intervention and a control group. Children and adolescents, who are eligible for participation, will be allocated in the control versus intervention group depending on the location (district) of their school to avoid situations where there are intervention and control participants in the same school/class. The evaluation will compare the resulting intervention and control group participants on all outcome variables. In addition, claims data on the CCSchool participants as well as on children and adolescents with the same inclusion diagnoses (a) living in districts not included in the study and (b) living in the CCSchool districts before CCSchool has started (historical comparisons) will be analysed. These external control participants are matched to the intervention participants by propensity score matching. The claims data allows validating the self-reported data on hospitalization rates and analysing potential effects on health care costs.

The statistical analyses of the comparisons between randomized control group, external control group and intervention group will use a multilevel framework taking the cluster randomisation into account. Longitudinal regression analyses of changes in hospitalization rates, quality of life, symptoms and absenteeism are conducted using mixed models adjusting for potential confounders (e.g., age, gender, regional characteristics). This approach is feasible to estimate within-person changes over time as well as group differences in within-change over time. For process evaluation, cross-sectional group comparisons will be analysed with regression models without repeated measures and descriptive statistics. All effects will be tested with a significance level of *p* ≤ .05.

To minimize selection bias, the study has been presented in each participating region to all child and adolescent psychiatrists and schools. CCSchool teams have invested high efforts in establishing collaborations between hospitals, health professionals in private practice and schools. The outcome variables to be collected are widely used instruments which have been scientifically extensively validated guaranteeing thereby highly standardized and reliable assessments.

## Discussion

CCSchool aims to improve collaboration and effectiveness of social, school and mental health services, by establishment of processes for early detection and diagnosis and implementation of treatment services in the natural environment. The study design assesses whether by this strategy the need for inpatient psychiatric care as well as resulting health care costs can be reduced. Results will show if the strategy of CCSchool can optimize care for children and adolescents with mental health problems by keeping them integrated in school, their peer groups and families, avoiding stigmatization and disintegration. If CCSchool proves to be effective it may become part of the regular benefit scheme of statutory health insurance in Germany.

## Abbreviations

CBCL: Child Behavior Checklist
CCST: CCSchool-Therapist
C-GAF: Child Global Assessment of Functioning
CIS: Columbia Impairment Scale
RT: Responsible Therapist
SDQ: Strengths and Difficulties Questionnaire
TRF: Teacher Report Form
YSR: Youth Self Report Form
